# Low doses of TiO_2_-polyethylene glycol nanoparticles stimulate proliferation of hepatocyte cells

**DOI:** 10.1080/14686996.2016.1239499

**Published:** 2016-10-17

**Authors:** Qingqing Sun, Koki Kanehira, Akiyoshi Taniguchi

**Affiliations:** ^a^ Cellular Functional Nanobiomaterials Group, Research Center for Functional Materials, National Institute for Materials Science, Tsukuba, Japan; ^b^ Graduate School of Advanced Science and Engineering, Waseda University, Tokyo, Japan; ^c^ Biotechnology Group, TOTO Ltd. Research Institute, Chigasaki, Japan

**Keywords:** TiO_2_-PEG NPs, low-dose exposure, cellular response, HGFRs aggregation, cell proliferation, 30 Bio-inspired and biomedical materials, 211 Scaffold / Tissue engineering / Drug delivery

## Abstract

This paper describes the effect of low concentrations of 100 nm polyethylene glycol-modified TiO_2_ nanoparticles (TiO_2_-PEG NPs) on HepG2 hepatocellular carcinoma cells. Proliferation of HepG2 cells increased significantly when the cells were exposed to low doses (<100 μg ml^–1^) of TiO_2_-PEG NPs. These results were further confirmed by cell counting experiments and cell cycle assays. Cellular uptake assays were performed to determine why HepG2 cells proliferate with low-dose exposure to TiO_2_-PEG NPs. The results showed that exposure to lower doses of NPs led to less cellular uptake, which in turn decreased cytotoxicity. We therefore hypothesized that TiO_2_-PEG NPs could affect the activity of hepatocyte growth factor receptors (HGFRs), which bind to hepatocyte growth factor and stimulate cell proliferation. The localization of HGFRs on the surface of the cell membrane was detected via immunofluorescence staining and confocal microscopy. The results showed that HGFRs aggregate after exposure to TiO_2_-PEG NPs. In conclusion, our results indicate that TiO_2_-PEG NPs have the potential to promote proliferation of HepG2 cells through HGFR aggregation and suggest that NPs not only exhibit cytotoxicity but also affect cellular responses.

## Graphical abstract

**Figure:**
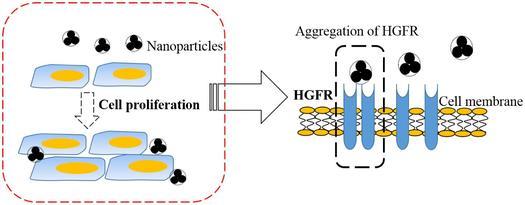

## 1. Introduction

TiO_2_ nanoparticles (TiO_2_ NPs) are utilized in a wide variety of industrial and consumer products, such as drug delivery systems,[1] antibacterial materials,[2] cosmetics,[3] sunscreens,[3] electronics,[4] and catalysts.[5] Consequently, various methods have been developed to prepare TiO_2_ NPs, including sol-gel techniques,[6] hydrothermal methods,[7] and solvothermal methods.[8] However, widespread use of TiO_2_ NPs has raised concerns about potential risks to human health, livestock, and the ecosystem due to long-term exposure and environmental release.[9]

In mammals, TiO_2_ NPs enter the body via gastrointestinal, dermal, or pulmonary absorption. The deposition mass of NPs is correlated with the exposure concentration. Koivisto et al. [10] found that the lung deposition mass increased though inhalation from 0.2 to 84.0 μg as the aerosol mass concentration increased from 0.8 to 28.5 mg m^–3^, showing a linear correlation with aerosol mass concentration. However, few NPs were deposited in the organs. Chen et al*.* [11] found that TiO_2_ NPs (80 nm) accumulated primarily in the liver of mice after oral administration, with a deposition mass of 3970 ± 1670 ng g^–1^. Another mouse study reported that the deposition mass of TiO_2_ NPs reached its highest level in the spleen, 1120 ± 880 ng g^–1^, after intraperitoneal injection for seven days.[12] These data suggest that it is more useful to study the effects of exposure to low doses of NPs at the cellular level. Many researchers have examined the cellular responses to exposure to high doses of NPs and found that high doses generate reactive oxygen species,[13] damage DNA,[14] and induce apoptosis,[15,16] inflammation,[17] and differentiation.[18] The cellular responses to exposure to low doses of NPs remain unclear, however.

PEG is generally considered a safe polymer and is therefore widely utilized in medicine and biotechnology due to its unique properties, such as biocompatibility, ready excretion from living organisms, and resistance to protein adsorption. In our work, PEG was used for functionalization of the TiO_2_ surface to decrease the cytotoxicity of NPs.

The aim of the present study was to characterize the cellular responses to exposure to low doses of TiO_2_-PEG NPs. For this purpose, HepG2 hepatocellular carcinoma cells were used. TiO_2_-PEG NPs were characterized by scanning electron microscopy (SEM) and an electronic light scattering. Cellular responses were evaluated and analyzed with respect to surface modifications. Our results show that TiO_2_-PEG NPs stimulate the proliferation of HepG2 cells through the aggregation of hepatocyte growth factor receptors (HGFRs), thus providing important information that enhances our understanding of nanotoxicology.

## 2. Materials and methods

### 2.1. Cell culture

HepG2 cells were cultured at 37 °C and 5% CO_2_ in Dulbecco’s modified Eagle medium (DMEM, high glucose, Nacalai Tesque, Kyoto, Japan) supplemented with 10% (v/v) heated fetal bovine serum (HFBS, Biowest, MO, USA), 100 μg ml^–1^ of penicillin, and 10 μg ml^–1^ of streptomycin (Nacalai Tesque). Cells were subcultured every two days.

### 2.2. Synthesis of NPs

TiO_2_ NPs were prepared as follows. Briefly, titanium (IV) oxide particles (anatase form containing rutile form) were purchased from Wako Pure Chemicals Industries (Osaka, Japan). Water-dispersed TiO_2_ particles were prepared by wet pulverization process under high pressure using Nanomizer^TM^ (NMS-200L, Nanomizer Inc., Kanagawa, Japan). Twenty ml of 25 wt% of the TiO_2_ particles in water was passed through the generator of Nanomizer 10 times under 200 MPa pressure, then water-dispersed TiO_2_ particles were collected. The surface of TiO_2_ NPs was then coated with PEG co-polymer as previously described.[19] The water-dispersed TiO_2_ particles were mixed with PEG-maleic acid copolymer (AM1510 K, Nihon Yushi Co., Ltd, Tokyo, Japan) modified with 4-amino-salicylic acid (Wako Pure Chemicals Industries). The final concentrations of these materials in dimethylformamide (DMF) were adjusted to 0.5 wt% of TiO_2_ and 1.5 mg ml^–1^ of polymer, respectively. Next, 20 ml of the mixture was incubated at 130 °C for 16 h, followed by complete drying at 40 °C for 10 min at a reduced pressure of 5 hPa. Thereafter, the TiO_2_-PEG NPs were re-dispersed in sterilized water at a concentration of 1 wt%.

### 2.3. Cell viability assay

The viability of HepG2 cells was assessed using a CellTiter-Glo® luminescent cell viability assay (Promega Corp., Madison, WI, USA) according to the manufacturer’s instructions. HepG2 cells were seeded at a density of 1 × 10^4^ cells/well in an opaque 96-well plate. After incubation at 37 °C and 5% CO_2_ for 24 h, the cells were exposed to TiO_2_ and TiO_2_-PEG NPs at concentrations of 0, 10, 20, 40, 80, 100, 400, and 1000 μg ml^–1^. At various times, the adenosine triphosphate (ATP) content of the cells was determined using a luminometer (TECAN, Tokyo, Japan) after adding an equal volume of CellTiter-Glo® reagent to each well.

### 2.4. Cell counting using the Trypan blue method

Nanomaterials may interfere with cell viability assays by light absorption, light scattering, or fluorescence.[20] To avoid or minimize NP-associated interference, cells were counted at various times using a disposable hemocytometer (Funakoshi, Tokyo, Japan). HepG2 cells were seeded at 1 × 10^5^ cells/well in 24-well plates and incubated at 37 °C and 5% CO_2_ for 24 h. The cells were then washed with phosphate buffered saline (PBS) once and exposed to 100 μg ml^–1^ of NPs for 12, 24, and 48 h. The cells were collected and stained with Trypan blue to distinguish dead and live cells; living cells were counted using a hemocytometer.

### 2.5. Cell cycle analysis

HepG2 cells were seeded in six-well plates at a density of 4 × 10^5^ cells/well. After incubation for 24 h, TiO_2_ and TiO_2_-PEG NPs were added to all wells except the control wells and incubated for 24 h. The cells were collected by trypsin treatment and then passed through a nylon mesh (Cell Strainer Snap Cap, Falcon, NY, USA) to remove cell clumps. Subsequently, the cells were washed twice with PBS and fixed with 66% ethanol at 4 °C for 2 h, after which they were washed twice with PBS and stained with 200 μl of 1 × propidium iodide (PI) and RNase staining solution (propidium iodide flow cytometry kit for cell cycle analysis, Abcam, Japan) and then incubated at 37 °C for 20 min. Finally, DNA content was assessed using a SP6800 spectral analyzer with 488 nm laser illumination to determine the cell phase.

### 2.6. Evaluation of cellular uptake by flow cytometry

To avoid artifacts associated with dye modification of the size of NPs, we used original NPs not exposed to dye to assess cellular uptake by HepG2 cells and determine the percentage of cells containing NPs.[21] Briefly, 2 ml of a HepG2 cell suspension at a density of 5 × 10^5^ cells/well was seeded in a six-well plate and cultured for 24 h. The attached cells were exposed to TiO_2_ and TiO_2_-PEG NPs at 0, 100, 200, 400, 600, and 800 μg ml^–1^. Ultrasonic processing of the NP suspensions for 30 min before and after dilution was employed to ensure adequate dispersion. After 24 h of incubation, cells were trypsinized and passed through a nylon mesh (Cell Strainer Snap Cap) after washing twice with PBS to remove excess NPs. The cells were then collected by centrifugation and suspended in 1 ml of PBS with 6% HFBS. Subsequently, the cells were stained with 1 μl of 42 μM PI (dead cells) and 2 μl of 4.3 mM thiazole orange (all cells) (BD Cell Viability Kit, BD Biosciences, Becton, Dickinson and Co., San Jose, CA, USA). Finally, stained cells were detected using an SP6800 Spectral Analyzer (Sony Biotechnology Inc., Tokyo, Japan). Cell granularity was assessed using side-scattering (SSC) light, and cell size was assessed using forward scattering light.

### 2.7. Immunofluorescence and confocal laser scanning microscopy

HepG2 cells were plated in a cell view cell culture dish (Greiner Bio-One North America, Inc., Monroe, NC, USA) at a density of 2.5 × 10^4^ cells/compartment and incubated for 24 h. Next, the cells were exposed to NPs at a concentration of 50 μg ml^–1^ and incubated for an additional 24 h, after which the cells were washed twice to remove excess NPs and then fixed with 4% paraformaldehyde (PFA) for 10 min. Subsequently, the cells were blocked with 1% bovine serum albumin (BSA)/10% normal goat serum/0.3 M glycine in PBS for 1 h, followed by washing three times (5 min each). Immediately after washing, the cells were incubated with anti–Met hepatocyte growth factor receptor (HGFR) antibody (1/100 dilution, EP1454Y, Abcam, Cambridge, MA, USA) for 1 h at room temperature (RT) and then goat anti-rabbit IgG H&L (1/200 dilution, DyLight® 488, ab96883, Abcam) in the dark for 1 h at RT. Both incubations were followed by washing three times (5 min each) with PBS. Confocal microscopy was performed using a confocal laser-scanning microscope (LSM510 META, Carl Zeiss Inc., Jena, Germany). All images were acquired using a 63 × 1.4 Plan-Apochromat oil immersion objective (Carl Zeiss).

### 2.8. Statistical analysis

All data were assessed for statistical significance using Student’s *t*-test. All values are presented as mean ± SD (*n* ≥ 3), *p ≤ 0.05, **p ≤ 0.01, ***p ≤ 0.001 which is typically provided only in the figure legends.

## 3. Results

### 3.1. Characterization of TiO_2_ and TiO_2_-PEG NPs

**Figure 1.**
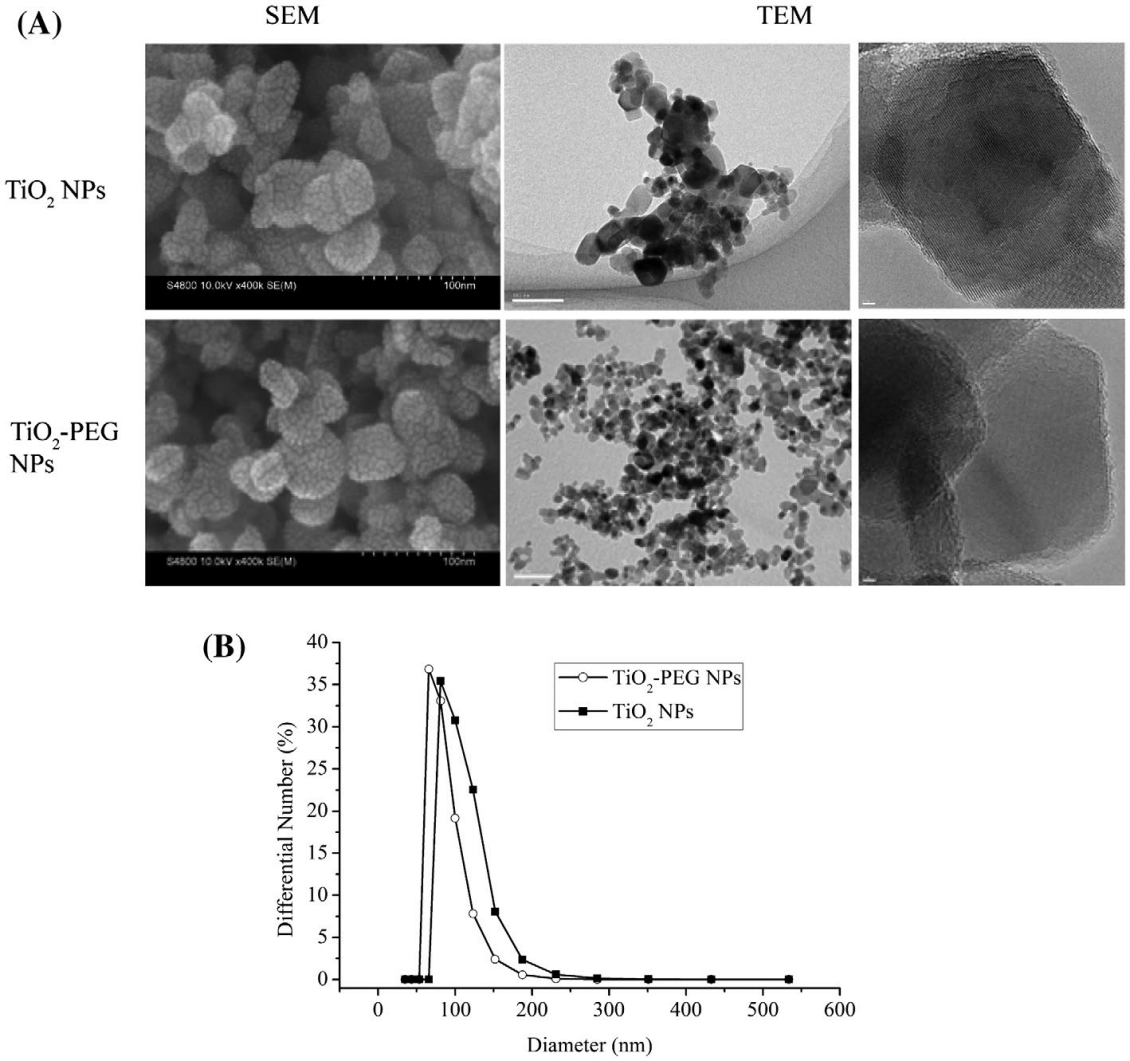
Morphology and size distribution of TiO_2_ and TiO_2_-PEG NPs in complete cell culture medium. (A) SEM and TEM images of TiO_2_ and TiO_2_-PEG NPs; (B) size distributions of TiO_2_ and TiO_2_-PEG NPs. Scale bar: 100 nm (SEM and middle TEM) and 2 nm (right TEM).

The morphology, size distribution, and dispersion of TiO_2_ and TiO_2_-PEG NPs in complete DMEM with 10% HFBS (cDMEM) were characterized by scanning electron microscope (SEM), transmission electron microscope (TEM) and electronic light-scattering detector (ELS). The results are shown in Figure 1. The SEM and TEM images indicated that the NPs were irregular and aggregated. The mean sizes of TiO_2_ and TiO_2_-PEG NPs were measured as 186 and 130 nm respectively in cDMEM. The polydispersity index (PDI) values of TiO_2_ and TiO_2_-PEG NPs were accordingly 0.224 and 0.149. Obviously, PEG modification induced the dispersion of TiO_2_ NPs easily and homogeneously.

Generally, when NPs are dispersed in culture medium, protein molecules adsorb onto the surface, forming a corona [22,23] that increases the mean size of the particles. However, surfaces covered with PEG resist protein adsorption due to the high steric exclusion of PEG.[24] In addition, PEG modification decreases the surface area/volume ratio and thus reduces the aggregation of NPs. Therefore, PEG modification was expected to reduce the tendency of TiO_2_-PEG NPs to aggregate.

### 3.2. Differences in cell viability at low and high NP doses

**Figure 2.**
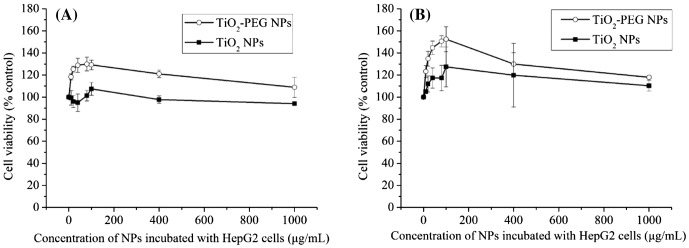
Growth of HepG2 cells after exposure to TiO_2_ and TiO_2_-PEG NPs for (A) 24 h and (B) 48 h. All values are presented as mean ± SD (*n* ≥ 3). Data were analyzed using Student’s *t*-test; *p ≤ 0.05, **p ≤ 0.01.

To determine the number of viable cells, a homogeneous assay was performed based on quantitation of cellular ATP content. As shown in Figure 2(A), within 24 h, cell viability of TiO_2_-PEG NPs increased in a dose-dependent manner from 100 to 150% at NP concentrations below 100 μg ml^–1^. Cell viability decreased to 110% as the dose of TiO_2_-PEG NPs was increased to 1000 μg ml^–1^. However, cell viability was still above 100%, indicating that TiO_2_-PEG NPs are not cytotoxic to HepG2 cells. The same trends were observed with an increase in incubation time to 48 h (Figure 2(B)), with cell viability slightly higher compared with 24 h of incubation. For TiO_2_ NPs, within 24 h, cell viability kept around 100% even when the NPs concentration increased to 1000 μg ml^–1^. With the incubation time increasing, cell viability increased slightly. The cell viability results showed that TiO_2_ and TiO_2_-PEG NPs have no cytotoxicity to HepG2 cells, and cells exposed to TiO_2_-PEG NPs have higher cell viabilities. Based on the observed increase in cell viability, an NP concentration <100 μg ml^–1^ was defined as a low dose, and correspondingly, concentrations ranging from 100 to 1000 μg ml^–1^ were defined as high doses due to the observed decreases in cell viability. In addition, the viability of cells exposed to TiO_2_-PEG NPs was significantly higher than that of control cells not exposed to NPs. These results thus suggest that at low concentrations, TiO_2_-PEG NPs promote cell proliferation.

Two different cell lines were examined in the viability assay, HepG2 and NCI-H292, although data are shown only for HepG2 cells. Only HepG2 cells exhibited significant proliferation, however. These results suggest that stimulation of proliferation is cell dependent.

### 3.3. Low concentrations of TiO_2_-PEG NPs induced cell growth

**Figure 3.**
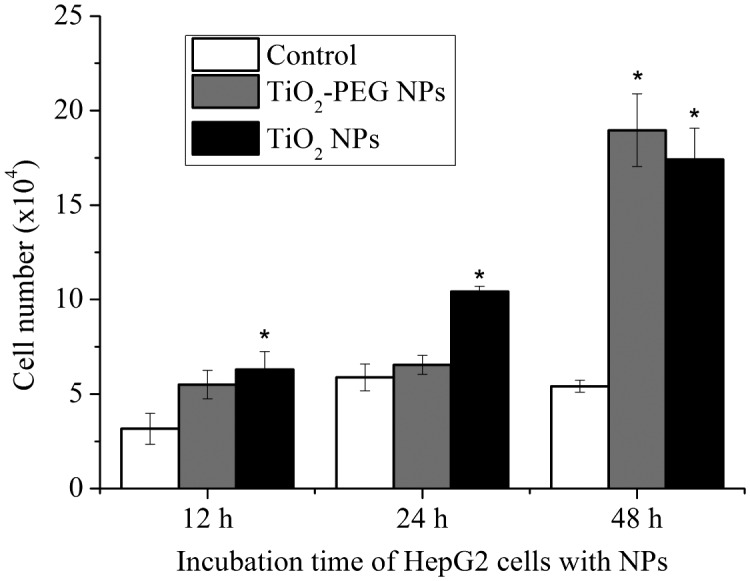
TiO_2_-PEG NPs induce proliferation of HepG2 cells. Cells were incubated without (white squares) or with TiO_2_ (black squares) and TiO_2_-PEG (gray squares) NPs at a concentration of 100 μg ml^–1^ for 12, 24, and 48 h. All values are presented as mean ± SD (*n* ≥ 3). Data were analyzed using Student’s *t*-test; *p ≤ 0.05, **p ≤ 0.01.

A cell counting assay was employed to verify the results of low-dose NP exposure. Live cells were stained with Trypan blue dye and counted. As shown in Figure 3, the number of HepG2 cells exposed to the low doses of TiO_2_-PEG NPs (≤100 μg ml^–1^) increased significantly at 12, 24, and 48 h compared with control cells not exposed to NPs. These results confirm that exposure to low doses of TiO_2_-PEG NPs promotes cell growth. TiO_2_ NPs did not increase cell numbers at 12 and 24 compared with control cells not exposed to NPs. However, after 48 h incubation cells exposed to TiO_2_ NPs showed significant growth, while control cells reached the confluent stage at approximately 24 h. That is because cells exposed to NPs seemed to grow with cell aggregation and overlay (data not shown). The results suggested that NPs that were outside of cells would connect cells and thus cause aggregation and overlay of cells.

### 3.4. Low concentrations of TiO_2_-PEG NPs induced changes in the cell cycle

To further examine the role of NPs in stimulating cell proliferation, we monitored the cell cycle by determining the DNA content of HepG2 cells using PI (a dye that stains nucleic acids) and flow cytometry analysis. As shown in Table 1, after exposure to TiO_2_-PEG NPs for 24 h, the DNA content of cells in the S phase increased significantly compared with control cells not exposed to NPs. However, for TiO_2_ NPs exposure group, the DNA contents of cells in S phase showed no significant increase compared with control. This result confirms that TiO_2_-PEG NPs enhance the ability of HepG2 cells to proliferate.

**Table 1.** Low doses of NPs induce DNA synthesis. HepG2 cells were incubated without (control) or with TiO_2_ and TiO_2_-PEG NPs at 100 μg ml^–1^ for 24 h. All values are presented as mean ± SD (*n* ≥ 3). Data were analyzed using Student’s *t*-test.

Group/24 h	Cell phases		
	G0/G1 (%)	S (%)	G2/M (%)
Control	55 ± 2	25.2 ± 0.5	19.4 ± 1.5
TiO_2_-PEG NPs	50.9 ± 0.7	29.5 ± 0.5*	19.6 ± 0.2
TiO_2_ NPs	55 ± 5	24 ± 3	19.8 ± 0.6

^*^ *p* ≤ 0.05

^**^ *p* ≤ 0.01.

### 3.5. Cellular uptake of TiO_2_ and TiO_2_-PEG NPs

**Figure 4.**
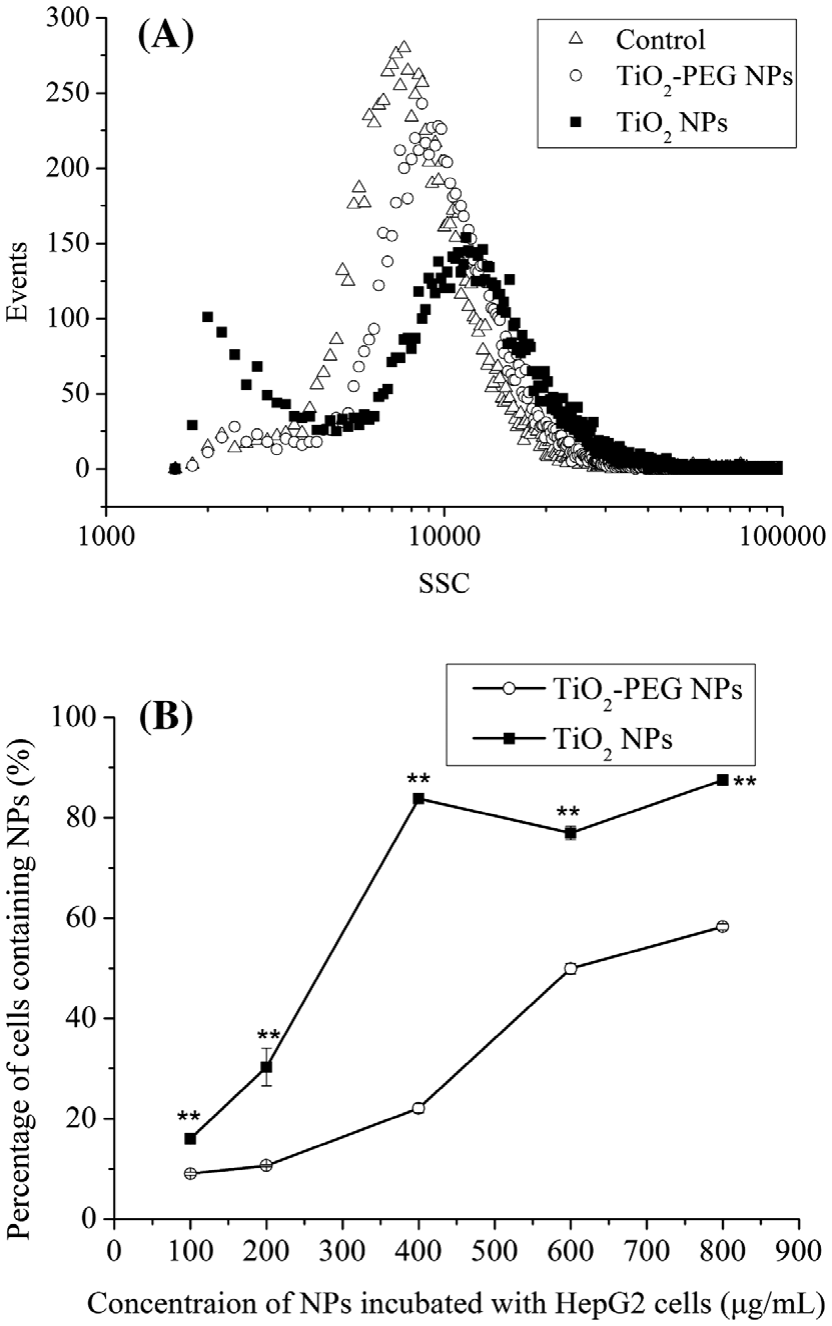
Cellular uptake of TiO_2_ NPs and TiO_2_-PEG NPs. (A) Uptake rate of NPs by HepG2 cells. Cells were incubated without (open triangles) or with (closed squares) TiO_2_ and (open circles) TiO_2_-PEG NPs at a concentration of 100 μg ml^–1^ for 24 h. (B) Percentage of HepG2 cells containing NPs. Cells were incubated with TiO_2_ (closed squares) and TiO_2_-PEG NPs (open circles) at 0, 100, 200, 400, and 800 μg ml^–1^ for 24 h. All values are presented as mean ± SD (*n* ≥ 3). Data were analyzed using Student’s *t*-test; *p ≤ 0.05, **p ≤ 0.01.

To elucidate the mechanism of cell growth promotion associated with exposure to low doses of TiO_2_-PEG NPs, cellular uptake of TiO_2_ and TiO_2_-PEG NPs was assessed by flow cytometry. As shown in Figure 4(A), compared with control cells, the SSC peaks of cells incubated with NPs shifted to the right, indicating NPs internalization. Figure 4(B) shows that the cellular uptake rate increased with increasing NPs dose. At NPs concentration of 100 μg ml^–1^, the percentage of cells containing TiO_2_-PEG NPs was the lowest, at only about 10%. The percentage of cells containing TiO_2_-PEG NPs increased to 60% at NPs concentration of 800 μg ml^–1^, however. Furthermore, compared with that of TiO_2_ NPs, TiO_2_-PEG NPs showed a much lower incorporation of cells, as shown in Figure 4(B). The cellular uptake rate was therefore lower at low doses of TiO_2_-PEG NPs, which decreased the cytotoxicity of NPs. Accordingly, high doses of TiO_2_-PEG NPs stimulated a higher cellular uptake rate, which resulted in higher cytotoxicity.

### 3.6. TiO_2_-PEG NPs induced aggregation of HGFRs

**Figure 5.**
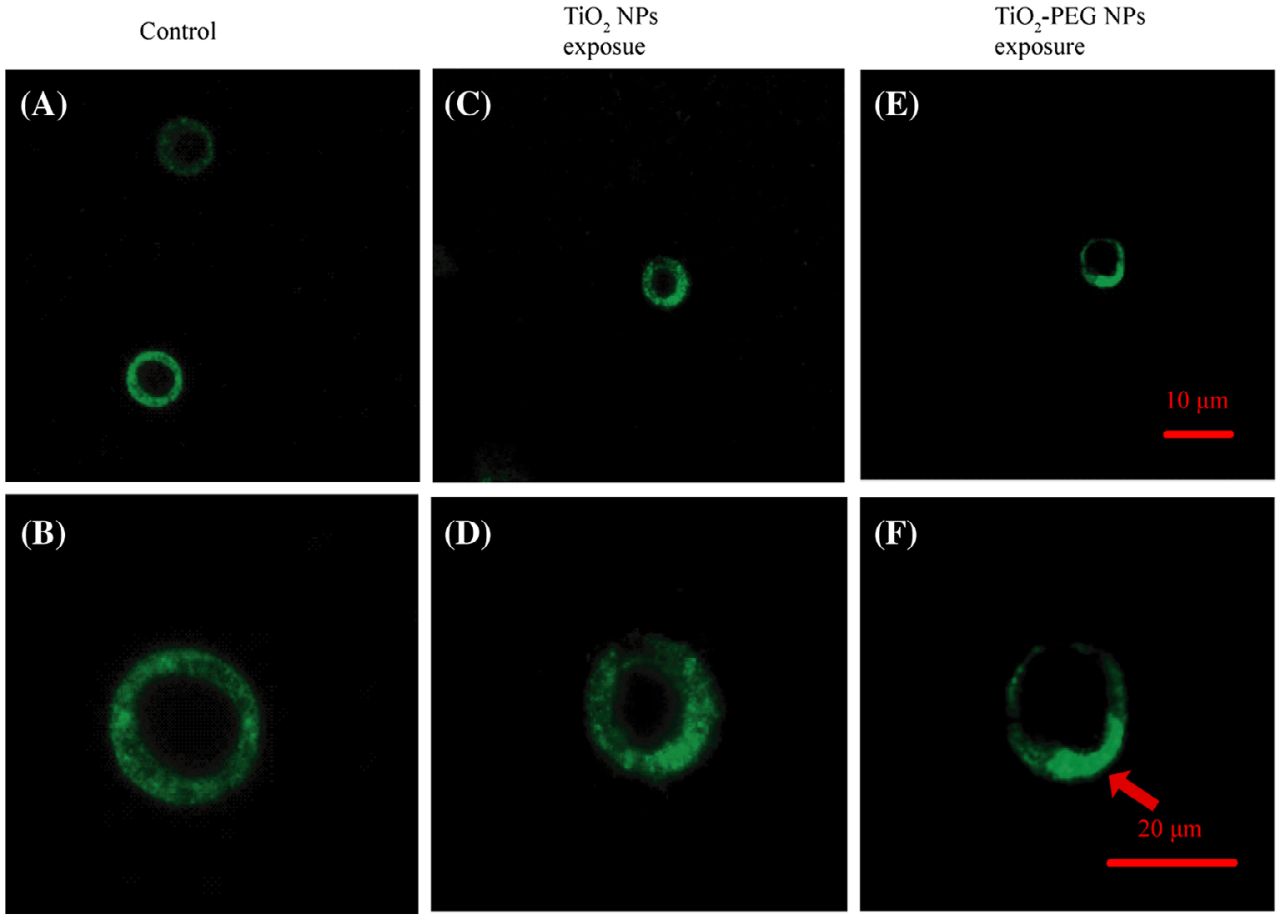
TiO_2_-PEG NPs induce the aggregation of hepatocyte growth factor receptors (HGFRs). Cells were incubated without or with TiO_2_ and TiO_2_-PEG NPs at 50 μg ml^–1^ for 24 h. (A, B) controls, without NPs exposure, the uniform distribution of the green fluorescence shows that HGFRs were scattered on the surface of cells; (C, D) TiO_2_ NPs exposure group: HGFRs were evenly scattered on the surface membrane of HepG2 cells; (E, F) TiO_2_-PEG exposure group: NPs induced HGFR aggregation, as shown by the arrow in F. Scale bar: 10 μm (A, C, E) and 20 μm (B, D, F).

The receptor for hepatocyte growth factor/scatter factor (HGF/SF) is a transmembrane tyrosine kinase encoded by the c-MET oncogene, which is known to mediate mitogenic and invasive responses in several cell types.[25,26] Recent research indicated that interactions between NPs and certain membrane receptors can promote aggregation of these receptors.[27] Therefore, we hypothesized that interaction of TiO_2_-PEG NPs with HGFRs would induce aggregation of HGFRs and subsequent proliferation of HepG2 cells. To test this hypothesis, the localization of HGFRs was assessed by immunofluorescence staining using an anti-HGFR antibody. As shown in Figure 5(A) and (B), HGFRs were scattered on the surface of control cells not exposed to NPs. Weak HGFRs aggregations on the surface of cells with TiO_2_ NPs exposure group were observed in Figure 5(C) and (D). HGFRs aggregated on the surface of cells exposed to TiO_2_-PEG NPs (as shown by the arrow in Figure 5(F)). These results confirmed that TiO_2_-PEG NPs exposure induces the aggregation of HGFRs in HepG2 cells, which in turn promotes cell proliferation.

## 4. Discussion and conclusions

In this work, the effect of TiO_2_-PEG NPs on HepG2 cells was examined. The low concentration of NPs was defined as less than 100 μg ml^–1^ of NPs in this work. The results of cell viability and cell counting assays confirmed that exposure to low doses (≤100 μg ml^–1^), even 10 μg ml^–1^, of TiO_2_-PEG NPs stimulates the proliferation of HepG2 cells. The significant increase in the DNA content of TiO_2_-PEG NP-exposed cells in the S phase also confirmed that exposure to low doses of TiO_2_-PEG NPs induces cell proliferation. To explore the mechanism through which exposure to low doses of TiO_2_-PEG NPs induces cell proliferation, we evaluated the cellular uptake of NPs at different concentrations. The TiO_2_ and TiO_2_-PEG NPs were used as non-fluorescence-labeled NPs for cellular uptake experiments to avoid the changes of size and surface characterization after modification. Therefore, in our results, there could be no artificial effects of different fluorescence intensities of NPs on the cellular uptake. We found that exposure to lower concentrations of TiO_2_-PEG NPs leads to lower rates of cellular uptake, which in turn decreases cytotoxicity. We also hypothesized that the observed increase in cell proliferation was related to a change in HGFR localization. Using immunofluorescence staining with an anti-HGFR antibody, we found that HGFRs aggregate on the surface of TiO_2_-PEG NP-exposed cells, confirming our hypothesis. Our results indicate that TiO_2_-PEG NPs induce the proliferation of HepG2 cells via promoting the aggregation of HGFRs and suggest that NPs exhibit not only cytotoxicity but also affect other cellular responses. We also investigated the same experiment using non-modified TiO_2_ NPs. TiO_2_-PEG NPs showed stronger effects that non-modified TiO_2_ NPs. The results suggested that both PEG-shell and TiO_2_ NPs themselves could be important for inducing cell proliferation by TiO_2_ NPs. PEG shell could change surface character and aggregation size of NPs, so these alterations of NPs might affect induction of cell proliferation.

**Figure 6.**
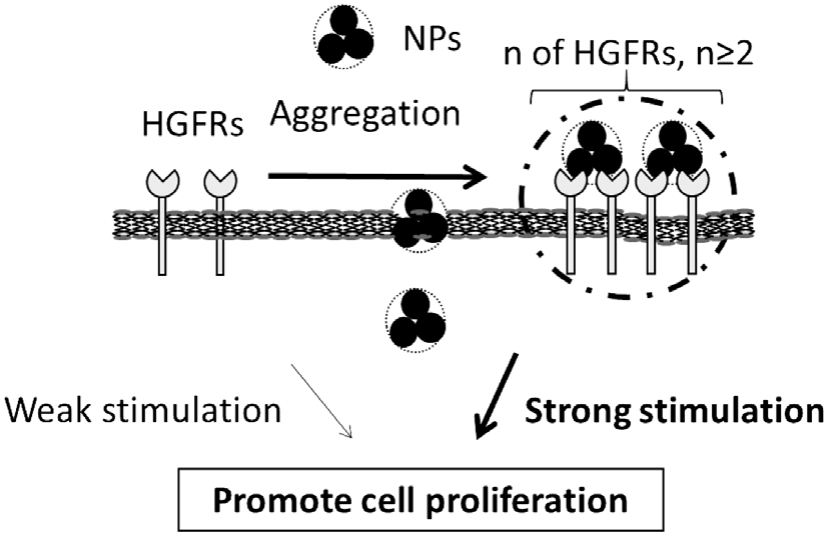
Potential mechanisms of cell proliferation stimulated by TiO_2_-PEG NPs. One hypothesis holds that insertion of TiO_2_-PEG NPs between the lipid bilayer changes the structure of the cell membrane, narrowing the space between HGFRs and leading to the formation of HGFR aggregates. The other hypothesis holds that TiO_2_-PEG NPs bind adjacent HGFRs together; ‘*n*’ indicates the number of HGFRs.

Details regarding how TiO_2_-PEG NPs interact with HGFRs and induce cell proliferation remain unknown. NPs can interact with cell receptors either directly or indirectly. Specific effects of direct interaction between NPs and membrane receptors include inducing the receptors to aggregate.[27] Alternatively, the protein coronas of NPs can interact indirectly with cell membrane receptors.[28] Based on our work, we propose two hypotheses to explain TiO_2_-PEG NP-induced cell proliferation, as illustrated in Figure 6. One hypothesis holds that insertion of TiO_2_-PEG NPs between the lipid bilayer changes the structure of the membrane, narrowing the space between HGFRs and leading to the formation of HGFR aggregates. The other hypothesis holds that TiO_2_-PEG NPs bind adjacent HGFRs together. These two hypotheses will guide our future efforts to elucidate the mechanism through which exposure to low doses of NPs induces cell proliferation. We understand that we did not fully explain this growth stimulation by NPs through HGFRs aggregation. So we tried blocking or knock-down experiments of HGFRs. However, these blocking or knock-down of HGFRs induced slow proliferation, even without NPs. Therefore, we could not show direct evidence for this growth inhibition.

## Disclosure statement

No potential conflict of interest was reported by the authors.

## References

[CIT0001] Zhang H, Wang C, Chen B (2012). Daunorubicin-TiO_2_ nanocomposites as a “smart” pH-responsive drug delivery system. Int. J. Nanomed.

[CIT0002] Etacheri V, Michlits G, Seery MK (2013). A highly efficient TiO_2-x_ C _x_ nano-heterojunction photocatalyst for visible light induced antibacterial applications. ACS Appl. Mater. Interfaces.

[CIT0003] Tucci P, Porta G, Agostini M (2013). Metabolic effects of TiO_2_ nanoparticles, a common component of sunscreens and cosmetics, on human keratinocytes. Cell Death Dis..

[CIT0004] Nunzi F, Agrawal S, Selloni A (2015). Structural and electronic properties of photoexcited TiO_2_ nanoparticles from first principles. J Chem Theory Comput.

[CIT0005] Ba-Abbad MM, Kadhum AAH, Mohamad AB (2012). Synthesis and catalytic activity of TiO_2_ nanoparticles for photochemical oxidation of concentrated chlorophenols under direct solar radiation. Int J Electrochem Sci.

[CIT0006] Macwan DP, Dave PN, Chaturvedi S (2011). A review on nano-TiO_2_ sol–gel type syntheses and its applications. J Mater Sci.

[CIT0007] Cheng WY, Deka JR, Chiang YC (2012). One-step, surfactant-free hydrothermal method for syntheses of mesoporous TiO_2_ nanoparticle aggregates and their applications in high efficiency dye-sensitized solar cells. Chem Mater.

[CIT0008] Mohan R, Drbohlavova J, Hubalek J (2013). Water-dispersible TiO_2_ nanoparticles via a biphasic solvothermal reaction method. Nanoscale Res. Lett.

[CIT0009] Long TC, Tajuba J, Sama P (2007). Nanosize titanium dioxide stimulates reactive oxygen species in brain microglia and damages neurons in vitro. Environ Health Perspect.

[CIT0010] Koivisto AJ, Mäkinen M, Rossi EM (2011). Aerosol characterization and lung deposition of synthesized TiO_2_ nanoparticles for murine inhalation studies. J Nanopart Res.

[CIT0011] Wang J, Zhou G, Chen C (2007). Acute toxicity and biodistribution of different sized titanium dioxide particles in mice after oral administration. Toxicol Lett.

[CIT0012] Chen J, Dong X, Zhao J (2009). In vivo acute toxicity of titanium dioxide nanoparticles to mice after intraperitioneal injection. J Appl Toxicol.

[CIT0013] Shukla RK, Sharma V, Pandey AK (2011). ROS-mediated genotoxicity induced by titanium dioxide nanoparticles in human epidermal cells. Toxicol In Vitro.

[CIT0014] El-Said KS, Ali EM, Kanehira K (2013). Effects of toll-like receptors 3 and 4 induced by titanium dioxide nanoparticles in DNA Damage-detecting sensor cells. J. Biosens. Bioelectron.

[CIT0015] Shukla RK, Kumar A, Gurbani D (2013). TiO_2_ nanoparticles induce oxidative DNA damage and apoptosis in human liver cells. Nanotoxicology.

[CIT0016] L’Azou B, Jorly J, On D (2008). In vitro effects of nanoparticles on renal cells. Particle and fibre toxicology. Part Fibre. Toxicol.

[CIT0017] Okuda-Shimazaki J, Takaku S, Kanehira K (2010). Effects of titanium dioxide nanoparticle aggregate size on gene expression. Int J Mol Sci.

[CIT0018] Liu X, Ren X, Deng X (2010). A protein interaction network for the analysis of the neuronal differentiation of neural stem cells in response to titanium dioxide nanoparticles.

[CIT0019] Yamaguchi S, Kobayashi H, Narita T (2011). Sonodynamic therapy using water-dispersed TiO_2_-polyethylene glycol compound on glioma cells: comparison of cytotoxic mechanism with photodynamic therapy. Ultrason. Sonochem.

[CIT0020] Monteiro-Riviere NA, Inman AO, Zhang LW (2009). Limitations and relative utility of screening assays to assess engineered nanoparticle toxicity in a human cell line[J]. Toxicol Appl Pharm.

[CIT0021] Matsuda S, Hitsuji A, Nakanishi T (2015). nduction of cell death in mesothelioma cells by magnetite nanoparticles. ACS Biomater Sci Eng.

[CIT0022] Dell’Orco D, Lundqvist M, Oslakovic C (2010). Modeling the time evolution of the nanoparticle-protein corona in a body fluid. PLoS ONE.

[CIT0023] Garvas M, Testen A, Umek P (2015). Protein corona prevents TiO_2_ phototoxicity. Plos One.

[CIT0024] Zhang M, Ferrari M, Gourley PL (1998). Reduction of protein adsorption on silicon coated with a self-assembled poly(ethylene glycol) and monomethoxypoly(ethylene glycol)..

[CIT0025] Di Renzo MF, Olivero M, Ferro S (1992). Overexpression of the c-MET/HGF receptor gene in human thyroid carcinomas. Oncogene.

[CIT0026] Natali PG, Nicotra MR, Di Renzo MF (1993). Expression of the c-Met/HGF receptor in human melanocytic neoplasms: demonstration of the relationship to malignant melanoma tumour progression. Br. J. Cancer.

[CIT0027] Marano F, Hussain S, Rodrigues-Lima F (2001). Nanoparticles: molecular targets and cell signalling. Arch Toxicol.

[CIT0028] Lynch I, Salvati A, Dawson KA (2009). Protein-nanoparticle interactions: what does the cell see?. Nat. Nanotechnol.

